# *In vitro* susceptibility to amphotericin B, itraconazole, voriconazole, posaconazole and caspofungin of *Aspergillus* spp. isolated from patients with haematological malignancies in Tunisia

**DOI:** 10.1186/2193-1801-3-19

**Published:** 2014-01-10

**Authors:** Soukeina Gheith, Fatma Saghrouni, Wadiaa Bannour, Yosra Ben Youssef, Abderrahim Khelif, Anne-Cécile Normand, Renaud Piarroux, Moncef Ben Said, Mansour Njah, Stéphane Ranque

**Affiliations:** Service d’Hygiène Hospitalière, CHU Farhat Hached, Sousse, 4000 Tunisie; Unité de recherche UR 04SP24, Ministère de la Santé Publique, Tunis, Tunisie; Laboratoire de Parasitologie -Mycologie, CHU Farhat Hached, Sousse, 4000 Tunisie; Service d’Hématologie Clinique, CHU Farhat Hached, Sousse, Tunisie; Parasitology & Mycology, CHU Timone-Adultes, Assistance Publique-Hôpitaux de Marseille, Marseille, 13005 France; Aix-Marseille Université, IP-TPT UMR MD3, Marseille, 13885 France

**Keywords:** Invasive aspergillosis, Haematological malignancies, *Aspergillus*, *In vitro* susceptibility, Antifungal drugs, Amphotericin B, Itraconazole, Voriconazole, Posaconazole, Caspofungin, Paradoxical growth, Trailing effect, MIC, *In vitro* susceptibility testing

## Abstract

The resistance of *Aspergillus* species to antifungal is increasingly reported and the knowledge of the local epidemiology and antifungal susceptibility pattern is pivotal to define adequate treatment policies. Our study aimed to: 1) describe the *in vitro* antifungal susceptibility profile of the *Aspergillus* species isolated from patients with haematological malignancies in Tunisia; 2) compare the E-test and Sensititre Yeast-One assays for the detection of paradoxical growth and trailing effect, both phenotypes commonly exhibited by *Aspergillus* spp. upon exposure to caspofungin and 3) to evaluate the mortality rate in patients according to the causative *Aspergillus* species and the antifungal treatment.

We tested amphotericin B, itraconazole, voriconazole, posaconazole and caspofungin against 48 *Aspergillus* isolates (17, *A. niger*; 18, *A. flavus*; 9, *A. tubingensis*; 1, *A. westerdijkiae*; and 1, *A. ochraceus*) with the E-test. Minimal inhibition concentrations were above the epidemiological cut-off values for amphotericin B in 67% of *A. flavus* strains; for caspofungin in 22% of *A. flavus* strains; and for itraconazole in 22% of *A. tubingensis* strains, voriconazole and posaconazole MICs were below the epidemiological cut-off values for all strains.

When exposed to caspofungin, 42% of the strains exhibited trailing effect and 38% paradoxical growth. Trailing effect occurred in 61% of *A. flavus* strains and paradoxical growth in 62% of *Aspergillus* section *Nigri* strains. E-test and Sensititre Yeast-One assays were only fairly concordant for the detection of these phenotypes. Repeatability of both assays was high for trailing effect but poor for paradoxical growth. The relatively high frequency of amphotericin B resistant strains makes voriconazole best adapted as a first-line treatment of invasive aspergillosis from amphotericin B to voriconazole in this hospital.

## Introduction

Invasive aspergillosis (IA) is a life threatening infection, especially in neutropenic patients where it is associated with a high mortality rate (Montagna et al., 2012; Blot et al., 2012). Over the last two decades, new antifungal agents including azoles and caspofungin were developed in order to improve the prognosis of IA. In parallel, tests for *Aspergillus* spp*. in vitro* antifungal susceptibility testing were developed and commercialized (Pfaller, 2012). The extensive use of antifungal agents was associated with the emergence of azole-resistant *Aspergillus* spp., and caspofungin (CS) has been recommended as a salvage treatment of IA (Pfaller et al., 2008; Jarque et al., 2013). Thus, the *in vitro* susceptibility testing of *Aspergillus* spp. clinical strains to antifungal agents is required both for driving and monitoring antifungal therapy and for the global surveillance of *Aspergillus* spp. susceptibility (Rex and Pfaller, 2002). *In vitro* susceptibility tests are based on the measurement of the fungal growth in the presence of different drug concentrations so as to determine the minimum inhibitory concentration (MIC) of antifungals (Wanger, 2012). Epidemiologic cut-off values (ECVs) of the MIC were established for different *Aspergillus* species and different antifungal agents, in order to assess the emergence of strains with decreased susceptibility (Espinel-Ingroff et al., 2011, Espinel-Ingroff et al. 2011b). A wild type organism being defined as a strain which does not harbor any acquired resistance to the particular antimicrobial agent being examined (Pfaller et al., 2011).

When exposed to CS, in addition to the clear end point phenotype (defined as the absence of growth at concentrations above the MIC), *Aspergillus* spp. exhibit two unusual *in vitro* testing phenotypes that are referred to as trailing effect (TE) and paradoxical growth (PG). TE is characterized by a reduced but persistent growth at concentrations above the MIC. PG is characterized by growth in the presence of low concentrations, no growth at intermediate concentrations, and growth resuming at higher concentrations (Fortwendel et al., 2010).

The aims of our study were to evaluate the *in vitro* antifungal susceptibility profile of *Aspergillus* spp. strains isolated from patients with haematological malignancies by using the E-test method, to assess both E-test™ and Sensititre Yeast-One™ (SYO) assays for the detection of PG and TE phenotypes exhibited by *Aspergillus* spp. upon exposure to CS and to evaluate the mortality rate in patients according to the causative *Aspergillus* species and the antifungal treatment.

## Material and methods

### Isolates

We tested 48 clinical *Aspergillus* isolates, including 17 *A. niger*, 18 *A. flavus*, 9 *A. tubingensis*, 2 *A. fumigatus*, 1 *A. westerdijkiae* and 1 *A. ochraceus*. These isolates were recovered from the sputa of 30 patients treated for acute leukaemia in the haematology unit of the Farhat Hached hospital of Sousse (central Tunisia). The sputa were inoculated onto Sabouraud-chloramphenicol medium and plates were incubated at 25 and 35°C. *Aspergillus* sections were identified on the basis of macroscopic and microscopic characteristics of the colonies (De Hoog et al., 2000), whereas the identification at the species level was performed by using both Matrix-Assisted Laser Desorption Ionization Time-Of-Flight (MALDI-TOF) mass spectrometry on a Microflex LT™ (Bruker Daltonics, Germany) instrument (Cassagne et al., 2012) and DNA sequencing of the ITS1-5.8-ITS2 and the 28S unit D1-D2 regions of the rRNA gene, and the partial beta-tubulin (BTUB) gene (Hendrickx et al., 2012).

### Antifungal susceptibility testing

We assessed the susceptibility of all the 48 isolates to the five following antifungal agents: amphotericin B (AMB), itraconazole (ITR), voriconazole (VOR), posaconazole (POS) and caspofungin (CS) by using the E-test (bioMérieux, France) assay with RPMI medium (AES, France) according to the supplier’s recommendations. The plates were incubated at 30°C for 48 hours. The MIC was determined at 100% inhibition for all tested antifungals and red at the lowest drug concentration at which the border of the elliptical inhibition intercepted the scale of the antifungal strip.

For each antifungal, we used the previously described ECVs, detailed in the Table 1, to detect the isolates within each species that might have acquired a mutational resistance mechanism to a given agent (Espinel-Ingroff et al., 2011, Espinel-Ingroff et al. 2011; Pfaller et al., 2011). Because ECVs had not been established for *A. tubingensis* (section *Nigri*) and for the *Circumdati* section, we used *A. niger*’s ECVs for *A. tubingensis* and those of *A. fumigatu*s for species of section *Circumdati*.

**Table 1:** **Epidemiological cut-off values for amphotericin, itraconazole, voriconazole, posaconazole and caspofungin according to the** ***Aspergillus*** **species**

Species	AMB ^a^ (mg/l)	ITR ^b^ (mg/l)	VOR ^c^ (mg/l)	POS ^d^ (mg/l)	CS ^e^ (mg/l)
***A. niger***	4	2	2	1	0.25
***A. flavus***	4	1	1	0.5	0.5
***A. fumigatus***	4	1	1	0.5	1
***A. tubingensis***	4	2	2	1	0.25
***Aspergillus*** **section** ***Circumdati***	4	1	1	0.5	1

^a^amphotericin; ^b^itraconazole; ^c^voriconazole; ^d^posaconazole; ^e^caspofungin.

We assessed both E-test and SYO (Trek Diagnostic Systems, Ltd., United Kingdom) assays for the detection of PG and TE phenotypes exhibited by *Aspergillus* spp. upon exposure to CS. Therefore, we analysed the CS *in vitro* phenotypes of 10 randomly selected isolates (4 *A. niger*, 4 *A. flavus* and 2 *A. tubingensis*) for which both E-test and SYO assays were performed in triplicate. The SYO was performed according the supplier’s recommendations. Briefly, the inoculum suspension was prepared from a 72 hours culture grown on Sabouraud dextrose agar and adjusted to the density of a 0.5 McFarland standard. The plates were incubated at 30°C and red at 24 and 48 hours by visual inspection. The MIC value corresponds to the first well that shows a change in colour from pink to purple (indicating inhibition of growth). ATCC 22019 and ATCC 6258 were used as quality control strains.

### Data analysis

MICs data of the E-test assay were presented as the MIC range, MIC_50_, and MIC_90_ for each species. The inter-assay concordance was estimated via Cohen’s kappa coefficient, by considering triplicate assays results as independent, using the following interpretation: no match [<0], poor agreement [0–0.2], fair agreement [0.21-0.4], moderate agreement [0.41-0.6], good agreement [0.61-0.8], very good agreement [0.81-1]. Logistic regression analysis was performed to calculate the risk of a patient’s fatal outcome associated with the first-line antifungal treatment. All statistical analyses were performed using SAS, version 9.2 (SAS Institute, Cary, NC).

This study was approved by the Comité d’Ethique et de Recherche de l’Hôpital Universitaire Farhat Hached de Sousse.

## Results

### Antifungal susceptibility

The MIC data are shown in Table 2. The MIC_50_ of all investigated antifungals for all *Aspergillus* species were low, indicating the absence of natural antifungal resistance of the tested species. When MIC_90_ are considered, AMB and ITR showed high values as compared to the remaining antifungals; thus indicating that some isolates had developed resistance to AMB and ITR.

**Table 2:** **MIC range, MIC50 and MIC90 of the 48 clinical** ***Aspergillus*** **isolates**

		MIC (mg/l)			
Species	Antifungal agent	Range	MIC _50_	MIC _90_	% > ECV ^f^
***A. niger*** **(n = 17)**	**AMB** ^**a**^	0.04–1.5	0.44	0.75	0
	**ITR** ^**b**^	0.38–2	0.98	2	0
	**VOR** ^**c**^	0.05–0.12	0.1	0.13	0
	**POS** ^**d**^	0.05–0.25	0.12	0.25	0
	**CS** ^**e**^	0.008–0.12	0.03	0.07	0
***A. flavus*** **(n = 18)**	**AMB**	0.5–0.32	6	20.8	66.6
	**ITR**	0.25–1	0.5	0.83	0
	**VOR**	0.06–0.5	0.19	0.25	0
	**POS**	0.06–0.25	0.19	0.25	0
	**CS**	0.004–32	0.064	32	22.2
***A. tubingensis*** **(n = 9)**	**AMB**	0.09–0.5	0.24	0.4	0
	**ITR**	0.25–8	0.19	4.8	22.2
	**VOR**	0.064–0.38	0.13	0.38	0
	**POS**	0.047–0.25	0.12	0.25	0
	**CS**	0.008–0.012	0.01	0.012	0
***A. fumigatus*** **(n = 2)**	**AMB**	3–6	NR^g^	NR	NR
	**ITR**	0.8–0.5	NR	NR	NR
	**VOR**	0.06–0.13	NR	NR	NR
	**POS**	0.06–0.13	NR	NR	NR
	**CS**	0.06–0.06	0.064	NR	NR
***Aspergillus*** **section** ***Circumdati*** **(n = 2)**	**AMB**	3–24	NR	NR	NR
	**ITR**	0.38–1	NR	NR	NR
	**VOR**	0.04–0.06	NR	NR	NR
	**POS**	0.09–0.25	NR	NR	NR
	**CS**	0.023–0.13	NR	NR	NR
**All species (n = 48)**	**AMB**	0.04–32	0.63	13.2	25
	**ITR**	0.25–8	0.5	2	4.1
	**VOR**	0.04–0.5	0.13	0.25	0
	**POS**	0.05–0.25	0.13	0.25	0
	**CS**	0.004–32	0.023	0.125	8.3

^a^amphotericin; ^b^itraconazole; ^c^voriconazole; ^d^posaconazole; ^e^caspofungin; ^f^epidemiologic cut-off value; ^g^not relevant.

The MIC_50_ and MIC_90_ of AMB were 0.63 and 13.2 mg/l, respectively, for all species. The MIC_90_ of AMB was much lower for both *A. tubingensis* (0.75 mg/l) and *A. niger* (0.4 mg/l) than for *A. flavus* (20.8 mg/l). Twelve (66.6%) *A. flavus* isolates had AMB MIC above the corresponding ECV, indicating that these isolates have acquired a resistance to AMB. All the 36 remaining investigated strains were susceptible to AMB.

The MIC_50_ and MIC_90_ of ITR were 0.5 and 2 mg/l, respectively, for all species. Only the black *Aspergillus* species had relatively high ITR MIC_90_: 2 mg/l for *A. niger* and 4.8 mg/l for *A. tubingensis*. Two (22%) *A. tubingensis* isolates had ITR MIC > ECV. The MIC of CS were > ECV in only 4 (22%) *A. flavus* isolates. The MIC of VOR and POS were below the ECVs in all the 48 tested isolates.

### Caspofungin *in vitro*susceptibility testing phenotypes

**Figure 1:**
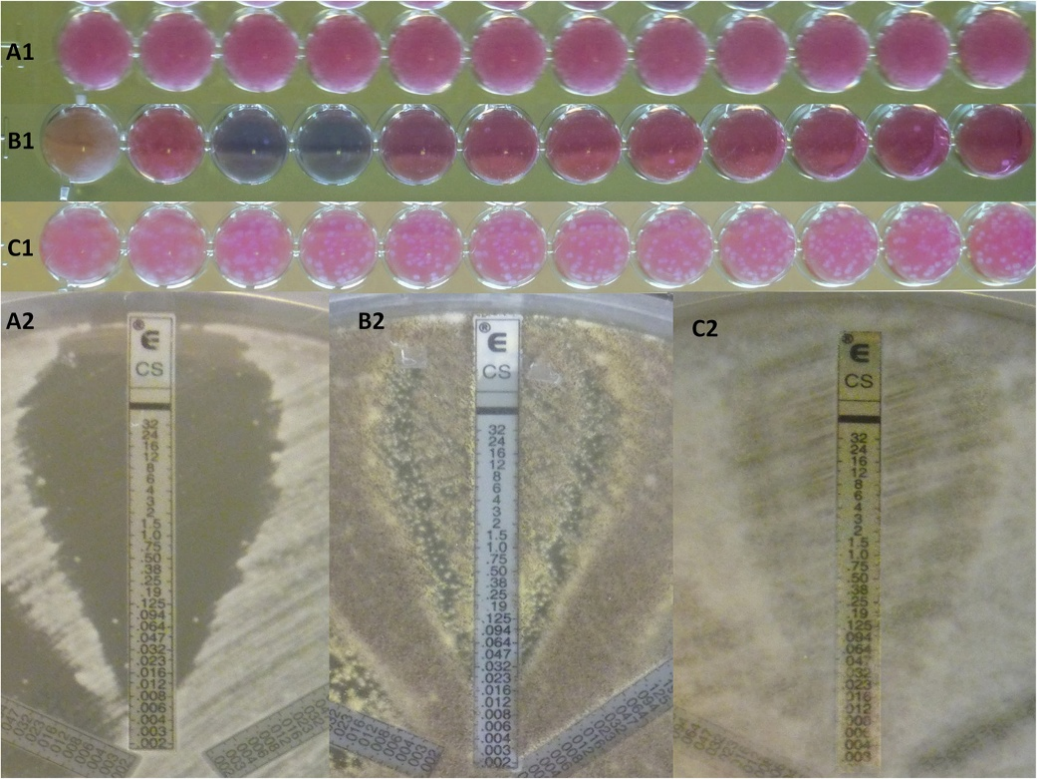
**Caspofungin** ***in vitro*** **testing phenotypes of** ***Aspergillus*** **spp. with the Sensititre Yeast-One (SYO, Trek Diagnostic Systems) and the E-test (bioMérieux) assays.** Clear end point (*A. tubingensis*, panel **A1**, SYO; panel **A2**, E-test), Paradoxical growth (*A. niger*, panel **B1**, SYO; panel **B2**, E-test) and Trailing effect (*A. flavus*, panel **C1**, SYO; panel **C2**, E-test).

In order to estimate the frequency of the different phenotypes exhibited by *Aspergillus* upon *in vitro* CS exposure, we considered the results of the E-test susceptibility profiles. The CS *in vitro* testing phenotypes are illustrated in Figure 1. Out of the 48 tested *Aspergillus* isolates, 10 (21%) exhibited a clear end point, 20 (42%) TE and 18 (38%) PG (Table 3). TE was more frequent with *A. flavus* (61%) and PG was only observed with the *Aspergillus* section *Nigri* (*A. niger* and *A. tubingensis*) isolates (61.5%). The mean CS concentration at which *Aspergillus* isolates growth resumed was 0.19 ± 0.15 mg/l.

**Table 3:** **Caspofungin E-test susceptibility testing phenotypes in 48** ***Aspergillus*** **isolates**

	Clear end point		Trailing effect		Paradoxical growth	
***A. niger*** **(n = 17)**	1	(6%)	4	(23.5%)	12	(70.5%)
***A. tubingensis*** **(n = 9)**	4	(44.5%)	1	(11%)	4	(44.5%)
***A. flavus*** **(n = 18)**	5	(28%)	11	(61%)	2	(11%)
***A. fumigatus*** **(n = 2)**	0		2	(100%)	0	
***A. ochraceus*** **(n = 1)**	0		1	(100%)	0	
***A. westerdijkiae*** **(n = 1)**	0		1	(100%)	0	
**All species (n = 48)**	10	(21%)	20	(42%)	18	(38%)

We further compared the repeatability of the different CS phenotypes with both E-test and SYO assays and estimated the inter-assay concordance. PG never occurred simultaneously with both E-test and SYO. The TE phenotype was highly repeatable with the *A. flavus* isolates especially when E-test was used, whereas PG phenotype was poorly repeatable using both assays (Table 4). E-test and SYO assays were fairly concordant with 0.35 and 0.26 kappa values for TE and PG, respectively.

**Table 4:** **Repeatability of triplicate caspofungin E-test and SYO** ***in vitro*** **susceptibility testing results**

Isolate	Species	E-test			SYO		
		Clear end point	Trailing effect	Paradoxical growth	Clear end point	Trailing effect	Paradoxical growth
1	*A. niger*	0	2	1	2	1	0
2	*A. niger*	0	2	1	1	0	2
3	*A. niger*	0	0	3	3	0	0
4	*A. niger*	1	1	1	2	0	1
5	*A. tubingensis*	1	1	1	1	0	2
6	*A. tubingensis*	1	1	1	2	0	1
7	*A. flavus*	0	3	0	0	3	0
8	*A. flavus*	0	3	0	1	2	0
9	*A. flavus*	0	3	0	0	3	0
10	*A. flavus*	1	2	0	2	1	0

### Mortality rate according to the *Aspergillus*species and antifungal treatment

Among the specimens obtained from the 18 patients who died, species of section *Nigri* were isolated from 10 (55.5%) sputa, species of section *Flavi* from 6 (33.3%) sputa and other *Aspergillus* species from 2 (11.1%) sputa (Table 5). The association of *Aspergillus* species with a fatal outcome was not statistically significant (p = 0.81). Among the patients with a fatal outcome, seven had been treated with AMB, two with VOR and nine received no antifungal treatment. The first-line antifungal treatment was statistically significantly (p = 0.02) associated with the patients’ outcome. Logistic regression analysis indicated that the risk of death was increased in the patients treated with AMB (OR = 3.89, 95% CI [0.80 to 18.98], p = 0.0144) and decreased in the patients treated with VOR (OR = 0.28, 95% CI [0.50 to 1.54], p = 0.0233) when compared to those who were not treated by antifungals.

**Table 5:** **Univariate analysis of the patients’ outcome according to the** ***Aspergillus*** **species and antifungal treatment**

		Death	Survival	P
***Aspergillus*** **section** ***Flavi***		6 (33%)	12 (40%)	0.81
***Aspergillus*** **section** ***Nigri***		10 (56%)	16 (53%)	
**Other** ***Aspergillus spp.***		2 (11%)	2 (11%)	
**Treatment**	**AMB**	7 (39%)	3 (10%)	0.02
	**VOR**	2 (11%)	12 (40%)	
	**No**	9 (50%)	15 (50%)	

## Discussion

Our study revealed that *Aspergillus* of sections *Nigri* and *Flavi* were the most frequently isolated species from our patients’ airways samples. This reflects the particular *Aspergillus* species spectrum involved in IA in Tunisia that contrasts with the prevalent data from developed Northern countries where *Aspergillus* section *Fumigati* is responsible for more than 80% of IA cases, followed by the section *Flavi* (10%); the sections *Nigri*, *Nidulantes* and *Terrei* causing the remaining 10% of cases (Krishnan et al., 2009; Wald et al., 1997). This finding may be explained by the qualitative and quantitative variations in environmental *Aspergillus* flora according to local climate and points out the need for *in vitro* susceptibility testing of clinical strains in order to select the appropriate predictive therapy.

All the tested strains of sections *Nigri*, *Fumigati* and *Circumdati* were susceptible to AMB with MIC_90_ < ECV. This finding is in accordance with the results of other studies where no AMB resistance was reported in isolates of the section *Nigri* (Hadrich et al., 2012a). Espinel-Ingroff et al. showed that there were no MIC above the ECV for *A. niger* (Espinel-Ingroff et al., 2011a). Similarly low AMB MIC (MIC mostly <0.5 mg/l) have been recently reported by Alcazar-Fuoli et al. and Baddley et al. for *A. niger* (Alcazar-Fuoli et al., 2009; Baddley et al., 2009).

In contrast, 66.6% of *A. flavus* isolates exceeded AMB’s ECV. This finding is in line with Hadrich et al., who showed that 84% of *A. flavus* strains isolated in Sfax, a city located south of Tunisia, had a reduced susceptibility to AMB (Hadrich et al., 2012b). It is also in accordance with those reported by Lass-Flörl et al. who showed that 67% of *A. flavus* isolates in Austria were resistant to AMB and that this *in vitro* resistance was associated with AMB therapy failure (Lass-Flörl et al., 1998). AMB resistance in *A. flavus* was of major concern because AMB deoxycholate was the first-line treatment of IA in our hospital at the time of the study.

ITR was found to be active on all tested *Aspergillus* species except for *A. niger* and *A. tubingensis. A. niger* showed a reduced susceptibility to ITR. The recently described *A. tubingensis* species was associated with higher ITR MICs as compared to *A. niger* (4.8 mg/l *vs* 2 mg/l) and MIC above ECV in 22% of isolates. The lower susceptibility of *A. tubingensis* strains might be related to the occurrence of a mutation, similarly to the one described by Howard et al. at position 97 in the CYP51A gene of *A. awamori*, another species of the section *Nigri* (Howard et al., 2011). The clinical impact of *in vitro* resistance to ITR of *Aspergillus* isolates is relatively modest because, especially in haematology patients, this antifungal agent is used for prophylaxis rather than for the curative treatment of IA (Döring et al., 2013).

With respect to VOR, neither resistance nor reduced susceptibility could be demonstrated in any of the strains tested. Our results contrast with those of Hendrickx et al. who reported higher MICs to both VOR and ITR in *A. tubingensis* (Hendrickx et al., 2012).

All strains were susceptible to POS, the MICs of which being as low as those of VOR. Our results contrast with those reported by Pfaller et al. who assessed the triazole ECVs of 1789 *Aspergillus* isolates and showed that the percentages of isolates for which MICs were greater than the ECVs ranged from 1.1 to 5.7% for POS, 0.0 to 1.6% for VRC and 0.7 to 4.0% for ITC (Pfaller et al., 2011).

More than 20% of *A. flavus* strains were resistant to CS. This finding contrasts with the one in all other *Aspergillus* species that were susceptible, with very low MICs, to CS. This relatively high resistance rate in *A. flavus* makes CS poorly adapted to the treatment of IA in the local hospital epidemiology setting. In contrast to our findings, Al-Wathiqi et al. showed that the MIC_90_ of *A. flavus* was 0.032 mg/l and MIC was above the ECV in 6% of *A. flavus* strains (Al-Wathiqi et al., 2013).

With respect to the *Aspergillus* species isolated from our patients’ samples, the low susceptibility of a significant proportion of strains to AMB and their high susceptibility to VOR, argue for the use of VOR as the first-line treatment of IA in our hospital, especially in the haematology unit. This is in line with the international recommendations (Seyedmousavi et al., 2013; Walsh et al., 2008).

By analysing the particular CS *in vitro* testing phenotypes, we concluded that TE and PG are not only dependent on the *Aspergillus* species, but also on the strain and the assay’s characteristics (Fortwendel et al., 2010). These distinct phenotypic responses to increasing CS concentrations might result from differences in the genetic background of *Aspergillus* species. Our study is the first to report the low level of agreement between E-test and SYO and the relatively poor reproducibility of both tests in the detection of TE or PG in *Aspergillus* spp. These findings argue against a simple genetic effect, and are more concordant with the hypothesis that the mechanism could be a compensatory up-regulation of the cell wall components’ synthesis, in response to high CS concentrations which stimulate chitin production (Fortwendel et al., 2010). Furthermore, the clinical relevance of the capacity of fungal subpopulations to survive and proliferate at high CS concentrations is debatable. Although the clinical significance of PG in fungi exposed to CS remains unclear, we were concerned by the fact that the concentration at which *Aspergillus* spp. growth resumed was clearly below the expected 1 mg/l plasma concentration in treated patients. Therefore, we suggest that CS would benefit of being combined with another antifungal in order to remove the PG effect (Gellen-Dautremer et al., 2010).

When the causative *Aspergillus* species was considered, the case fatality rate was similar between our patients. In contrast, a significant association between the outcome of the patients and the antifungal treatment was demonstrated. This association should however be cautiously interpreted, mainly because the treatments were not randomly allocated. The fact that VOR was associated with a better outcome is in agree with the current recommendations to using VOR as the first-line treatment of IA (Walsh et al., 2008). The higher case fatality rate in the patients who were treated with AMB as compared to those who did not receive any antifungal treatment might result from the drug’s toxicity or from a more severe form of IA disease in treated patients. Furthermore, it is well known that the clinical outcome does not only depend on the *in vitro* antifungal susceptibility profile, but also on host factors, including underlying disease, cellular and humoral immune functions and antifungal agent’s pharmacokinetic/pharmacodynamic properties; all these factors are considered to play a critical role in the patients’ response to the treatment (Steinbach et al., 2003).

## Conclusions

*Aspergillus* of the section *Nigri* and *Flavi* were the most frequently involved in IA in our patients with acute leukaemia. More than 2/3 of *A. flavus* isolates showed a reduced susceptibility to AMB, and 22% of *A. tubingensis* showed a reduced susceptibility to ITR. Based on these findings, we recommend VOR for the first-line treatment of IA in this haematology unit. The relatively poor reproducibility of CS *in vitro* testing results suggests that it cannot reliably be used to predict the patients’ outcome. Further studies aiming at determining the clinical significance of both TE and PG *in vitro* phenotypes are warranted, mainly because when they occur, *Aspergillus* spp. show the capacity to grow at concentrations clearly below the expected plasma drug concentration in treated patients.

## Abbreviations

SYO: Sensititre Yeast-One
MIC: Minimum inhibitory concentration
ECV: Epidemiologic cut-off values
AMB: Amphotericin B
POS: Posaconazole
VOR: Voriconazole
ITR: Itraconazole
CS: Caspofungin
TE: Trailing effect
PG: Paradoxical growth
OR: Odds-ratio.
